# High-Resolution Graphite Furnace Atomic Absorption Spectrometry Determination of Bismuth in Lithium Niobate Optical Crystals

**DOI:** 10.1021/acsomega.5c05486

**Published:** 2025-10-05

**Authors:** Dániel Csontos, László Kovács, Krisztián Lengyel, László Bencs

**Affiliations:** Department of Applied and Nonlinear Optics, Institute for Solid State Physics and Optics, HUN-REN Wigner Research Centre for Physics, P.O. Box 49, H-1525 Budapest, Hungary

## Abstract

High-resolution continuum source graphite furnace atomic absorption spectrometry (HR-CS-GFAAS) methods with solid and solution sampling were developed for the determination of the Bi dopant in high-purity lithium niobate (LiNbO_3_) crystals. Samples were cut from the cylindrical crystal bulks, cleaned, and pulverized. A HF–HNO_3_ mixture was applied for microwave digestion of LiNbO_3_ (≈0.07 g per sample). Atomization transients, pyrolysis, and atomization curves were studied with various media and chemical modifiers, e.g., triammonium citrate (TAC), Pd–Mg­(NO_3_)_2_. For solid samples, the optimal pyrolysis and atomization temperatures of Bi were found at 1000 and 1800 °C, respectively, whereas, for solution samples, much lower values of 500 and 1300 °C were obtained. TAC slightly, but Pd–Mg considerably increased the optimal pyrolysis and atomization temperatures, i.e., up to 1300 and 2100 °C, respectively. The dissolved LiNbO_3_ matrix acted as an internal modifier, exhibiting optimal pyrolysis and atomization temperatures for Bi. For solid sample analysis, 0.05–0.4 mg (average: 0.1 mg) of LiNbO_3_ powder was dosed into graphite boats, while conventional standard addition and three-point estimation were used for calibration. The limit of detection (LOD) was 0.4 and 0.3 μg/g for solid and solution analysis, respectively, using Bi I 227.6580 nm and Bi I 223.0608 nm lines, respectively. Utilizing the latter for solid sampling, an LOD of 0.03 μg/g can be attained. The analytical results for all methods were in good agreement (mean bias: <12%). The precision of the solid and solution sample methods was at 6–16% (average: 12%) and 1–13% (average: 4.4%), respectively. The Bi content of the crystals ranged from 56 to 311 μg/g. The characteristic mass for solid and solution sampling was 220 and 17 pg, respectively. The accuracy of the method was checked against the GBW07407 (laterite soil) certified reference material.

## Graphical abstract

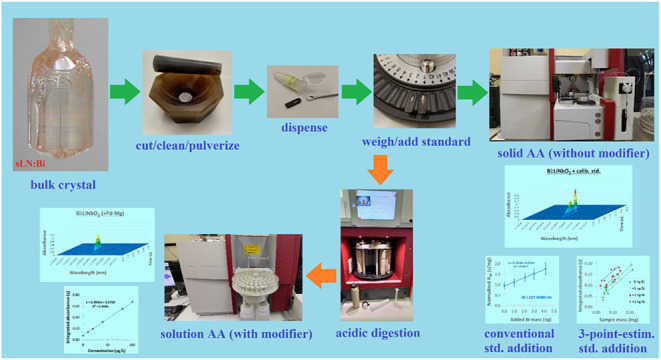

## 1 Introduction

High-purity lithium niobate (LiNbO_3_) crystals are attractive hosts for nonlinear optical applications, and therefore, are the subject of extensive physical–chemical studies.^1–3^ These crystals are usually grown from melts of metal oxides using the Czochralski and the high-temperature top-seeded solution growth (HTTSG) methods.^3^ The optical properties of the crystal can be tuned with specific dopants, such as Bi, Fe, and Mg, added as oxides to the starting materials before crystal growth.^4–6^ Among these elements, Bi is an outstanding dopant, exhibiting real-time holographic display^5^ and long-lived photochromic signals^6^ in lithium niobate. Bismuth is characterized by a small segregation coefficient, i.e., low build-in percentage in LiNbO_3_.^6^ Additionally, the segregation of Bi along the crystal growth axis may deviate from homogeneous; thus, it is important to accurately quantify its vertical concentration distribution in the crystal bulk for research and practical applications. Bismuth-based materials can also play a role in environmental remediation, for example, Bi nanoparticles loaded into porous carbon nanofibers facilitate CO_2_ conversion.^7^ Bi_2_Fe_4_O_9_ piezo-catalytically activates peroxydisulfate to degrade organic pollutants.^8^ Consequently, the determination of Bi in these material systems is important, which requires analytical methods of high sensitivity and selectivity.

Atomic absorption spectrometry (AAS) and inductively coupled plasma (ICP) based techniques usually provide a suitable analytical platform for the determination of impurities and dopants in oxide-based optical crystals.^9–13^ Examples of such analyses include the determination of Cr,^9^ Fe,^9,10^ Si,^10^ Mg,^11^ Mn,^9^ Li,^12,13^ Nb,^12^ and V^13^ in LiNbO_3_, for which solid sample and solution introduction methods were applied. The latter involved acidic dissolution of samples in high-pressure bombs in an oven,^9,10^ or in open platinum crucibles, exposed to a roaring blue Bunsen flame.^9^ So far, Bi has been quantified in a couple of material systems by means of furnace AAS techniques,^14–31^ as summarized below, but not yet characterized in LiNbO_3_. Peculiarities in the determination of Bi in environmental matrices using various analytical chemical techniques can be found in a comprehensive literature review.^32^

Bismuth, being a volatile element, has been extensively studied in line-source (LS) furnace AAS, particularly using various kinds of chemical modifiers, with the specific aim of thermally stabilizing the analyte in the atomizer.^33–40^ In an early study, Schlemmer and Welz^33^ applied Pd–Mg­(NO_3_)_2_, being referred to as a universal modifier, for a number of elements including Bi. They observed a pyrolysis temperature (*T*_pyr_) of 1200 °C as optimum for Bi, while the atomization temperature (*T*_at_) of Bi reached its optimum at ≈2000 °C. Interestingly, Ni and Shan^34^ proposed Pd alone as a universal modifier in graphite furnace atomic absorption spectroscopy (GFAAS) for volatile analytes, including Bi. Slaveykova et al.^35^ suggested the use of Ir as a permanent modifier, deposited on W-treated, integrated graphite platform (IGP) of the transversely heated graphite atomizer (THGA) for Bi quantitation, achieving *T*_pyr_ of 1100 °C, or even 1400 °C using Ir modifier alone. Barbosa et al.^36^ applied W–Rh coating on IGP of the THGA for the determination of Bi in human urine and blood samples, to increase the optimal *T*_pyr_ to 1100 °C. Dobrowolski et al.^37^ studied carbides of Nb, Ti, W, and Zr as permanent modifiers to augment the Bi signal in slurry sampling GFAAS analysis of environmental samples and certified reference materials (CRMs). NbC proved to be the most efficient modifier. While Ba­(NO_3_)_2_ was applied as a chemical modifier to surpass matrix interferences arising from covaporization of the high sulfur content of the samples. Tokman and Akman^38^ investigated the mechanism of CoCl_2_ matrix on the vaporization and atomization of Bi in GFAAS, using a dual-cavity graphite platform, which allowed the distinction between gas-phase and condensed-phase interferences. Colloidal Pd–Mg applied as nitrates acted concurrently as analyte and matrix modifiers. Narukawa et al.^39^ also investigated CoCl_2_ as a chemical modifier to stabilize Bi, but on a tungsten boat in tungsten furnace AAS. They reached as high optimal *T*_pyr_ as 1450 °C. Pszonicki and Dudek^40^ studied Pd, Mg, and Pd–Mg for the determination of Bi in standards stabilized with HNO_3_ or HCl. Pd acted properly in HNO_3_ media in terms of thermal stabilization of Bi up to 1100 °C, whereas lower optimal *T*_pyr_ was observed for HCl media. Furthermore, Mg and Pd–Mg modifiers were reported to be quite ineffective for Bi. On the other hand, Irwin et al.^41^ analyzed single-alloy chips (mass: 0.5–4 mg) to determine impurities, using the stabilized temperature platform furnace technology, and concluded that the Ni alloy as a modifier acted better than reduced Pd for Bi. Further peculiarities on the mechanisms of action of the most important chemical modifiers applied in GFAAS can be found in extensive reviews of Ni and Shan,^34^ Tsalev et al.,^42^ Ortner et al.,^43^ and Volynsky.^44^

In this study, solid and solution sample introduction high-resolution continuum source GFAAS (HR-CS-GFAAS) methods were elaborated and applied for the quantitation of Bi in samples of high-purity, stoichiometric LiNbO_3_ (sLN) crystals. Atomization transients, pyrolysis and atomization curves, and calibration methods were studied with the use of various sample media and chemical modifiers such as triammonium citrate (TAC) and Pd–Mg­(NO_3_)_2_.

## 2 Experimental Section

### 2.1 Instrumentation

The experiments were performed on an Analytik Jena Model ContrAA-700 (Jena, Germany) tandem HR-CS-AAS spectrometer, equipped with a THGA. The analysis was supported by an SSA-600L (Analytik Jena) automatic solid sampler fitted with a microbalance (resolution: 1 μg) and a liquid dosing accessory (LDA). Calibration standards were dispensed using LDA or manual pipetting (Brand Transferpette). For solution analysis, samples and standards were dispensed with an MPE-60 autosampler (Analytik Jena).

For AAS measurements, a high-pressure xenon arc lamp operating in hot-spot mode with a current of 13 A served as the primary light source. The spectrometer optics encompass an HR-monochromator, incorporating a predispersive prism and an Echelle grating, arranged in Littrow-mounting. The dispersed UV–vis spectra are imaged on a 588-pixel charge-coupled device (CCD). Of these, 200 pixels are utilized for the detection of the selected spectral range, while the rest serve as internal corrections. The optics provide high-resolution spectra in the picometer (pm) range. The resolution decreases slightly with increasing wavelength, due to nonlinear prism predispersion.

For solid sample analysis, pyrolytic graphite-coated orificeless graphite IC-tubes (Analytik Jena, Part No. 407-A81-303) with electrographite sample insertion boats were utilized. These boats with an LWH-size of about 14.2 × 4.5 × 2.1 mm (see TOC graphic) were fabricated in the mechanical workshop of the host institute from high-density, spectrally pure electrographite rods (diameter: 6 mm, length: 200 mm, type: SW-104, Kablo Bratislava, Topoľčany, Slovakia). For solution analysis, pyrolytically coated THGA tubes fitted with PIN-platforms (Analytik Jena, Part No. 407-A81-025) were applied. Graphite tubes were preconditioned three times daily before the start of analysis. High-purity (5 N) argon (Messer-Hungary, Budapest) flowed as a sheath gas in and around the graphite atomizer. The lifetime of THGA graphite tubes tested with LiNbO_3_ matrix fell into the range of 300–350 analytical cycles, whereas that of graphite boats used for solid samples was between 50 and 70 firings. The optimized THGA heating programs for the analysis of solid and solution samples are given in Table 1.

**Table 1:** Graphite Furnace Heating Programs for Bi Determination in Lithium Niobate and Soil CRM Samples

step	temp. (°C)	ramp (°C/s)	hold time (s)	gas flow rate (L/min)
drying-1	80	6	20	2
drying-2	90	3	20	2
drying-3	110	5	10	2
pyrolysis-1	350	50	20	2
pyrolysis-2	500^a^, 900^b^, 550^c^ ^,^ ^d^	300	20^a^ ^,^ ^b^, 10^c^	2
gas adaption	as pyrolysis-2	0	5	0
atomization	1900^a^ ^,^ ^c^, 2100^b^, 2300^d^	1500^a^ ^,^ ^c^, 2000^b^, 2500^d^	3^a^, 7^b^, 8^c^ ^,^ ^d^ ^,^ ^e^	0
cleaning	2450^a^ ^,^ ^b^ ^,^ ^c^, 2600^d^	500	4	2

a Without modifier/matrix-matching/with TAC modifier.

b With 5 μg Pd + 1.0 μg Mg (nitrate) modifier (solution analysis).

c Solid sampling.

d CRM sample (with Pd–Mg modifier).

e Signal integration times of 3.9, 7.6, and 8.9 s, respectively.

For solid sample and solution analysis, the spectral lines of Bi I 227.6580 and Bi I 223.0608 nm were applied, respectively. The former possesses a relative strength of 7% compared to the latter, the primary analytical line. At these wavelengths, the resolution of the spectrometer (per CCD pixel) is 1.38 and 1.42 pm, respectively. For signal evaluation, the peak volume selected absorbance (PVSA) was chosen, standing for integrated absorbance (*A*_int_) of the central pixel (CP) and adjacent pixels (CP ± 1). The method of iterative background (BG) correction was utilized to compensate for nonspecific absorption. Analytical lines and molecular bands were assigned using the spectral database of the ContrAA-spectrometer (ASpect Software, version: 2.1.2.0., Analytik Jena AG). Alternatively, the NIST Atomic Spectral Data Base,^45^ the spectral tables of Zaidel’ et al.^46^ and Pearse and Gaydon^47^ were utilized.

### 2.2 Materials and Methods

The reagents were at least of analytical grade (a.g.) or better (suppliers: Merck, Darmstadt, Reanal, Budapest). High-purity, deionized water (18.2 MΩ cm), obtained from a Millipore SAS Elix Essential (UV) water purifier (Merck), was applied for standard and sample preparation and dilution. Seven to nine standard solutions were diluted from a stock solution of Bi (1000 mg/L, Merck). Additionally, Multielement ICP-Standard Solution IV (Merck) was used after proper dilution to check the accuracy of the calibration standards.

The growth of the boules of doped (Bi, Bi+Mg) and undoped stoichiometric LiNbO_3_ single crystals is described in detail elsewhere,^4^ and the sample cuts as well.^6^ Each crystal sample was cleaned in a 1:1 mixture of abs. ethanol–acetone for an hour and washed afterward with Milli-Q water. After that, the samples were parched at ≈80 °C in a Binder Model ED-53­(E2) electric drying oven (Binder GmbH, Tuttlingen, Germany). The dried crystal samples were pulverized in an agate mortar with an agate pestle. These utensils were precleaned before pulverization of each sample.

For solid sampling, 0.05–0.4 mg of the powdered sample was dispensed into the sample insertion boat and weighed on the microbalance of the SSA-600L. Then, the boat was moved into the graphite furnace for measurement, using the robotic arm of the SSA-600L. The masses of some samples were checked on a Sartorius Model SE-2 ultramicrobalance (Göttingen, Germany, resolution: 0.1 μg). No significant difference between the readings of the two microbalances was observed. For solution analysis, a 20 μL aliquot of the sample or the standard was dispensed onto the PIN platform of the THGA tube, while 5 μL of the Pd–Mg­(NO_3_)_2_ modifier was dosed along with the sample or standard. The modifier was prepared by mixing and diluting 10 g/L Pd­(NO_3_)_2_ and 10 g/L Mg­(NO_3_)_2_ solutions (Merck) in an optimal 5:1 ratio (Figure S1). A 1.0 mol/L stock solution of the TAC modifier was prepared from stoichiometric amounts of citric acid powder (e.g., Reanal, Budapest, Hungary) and ammonia solution (25%, Suprapur, Merck).

For the preparation of solution samples, the LiNbO_3_ powders were decomposed using an Anton Paar Multiwave 5000 microwave digestion system (MWDS) equipped with an 8NFX rotor. First, ≈0.07 g of the sample was weighed in the polytetrafluoroethylene (PTFE) digestion vessel of the MWDS by using a Kern-770 analytical balance (Kern-Sohn GmbH, Balingen, Germany). A mixture of 5 mL of 8 + 8 mol/L HF + HNO_3_ (Merck, Suprapur) was added to each sample. The MWDS-program consisted of heating (10 min, 1350 W), holding at temperature (25 min, 1350 W), cooling (10 min, 0 W), and ventilation (15 min, 0 W) steps. Three replicates were made for each sample solution with a final volume of 50 mL. Samples with higher Bi content were diluted 5-fold for analysis.

### 2.3 Calculation Methods and Statistical Analysis

The measurement data was processed using Microsoft Excel. The arithmetic mean, standard deviation (SD), and relative standard deviation (RSD) were calculated from triplicate analytical cycles for solution analysis (*n* = 3). For solid sample analysis, due to the higher within-run variation, a larger number of consecutive measurements (*n* = 10–15) were performed, to increase the confidence of the determinations. The calibration curves were fitted to the calibration points with the least-squares method. Outliers were checked with Nalimov’s test^48^ and omitted from further evaluation. The *F*-test and Student’s *t*-test were applied to compare the analytical results. A confidence level of *p* = 0.05 or 0.01 was used. The limit of detection (LOD) data was calculated from the average response (background equivalent concentration) of the blanks (*n* = 11) plus 3 × SD/*S*_c_ (where *S*_c_—slope of calibration). Solid and solution blanks were prepared with undoped (nominally pure) LiNbO_3_ samples. The limit of quantitation (LOQ) data was evaluated analogously, but involving 10 times the SD of the blank.

## 3 Results and Discussion

### 3.1 Pyrolysis and Atomization Curves

The pyrolysis and atomization curves, calculated for sample mass normalized absorbance (*A*_int‑n_), were recorded by dosing the Bi-doped LiNbO_3_ powder (Figure 1).

**Figure 1:**
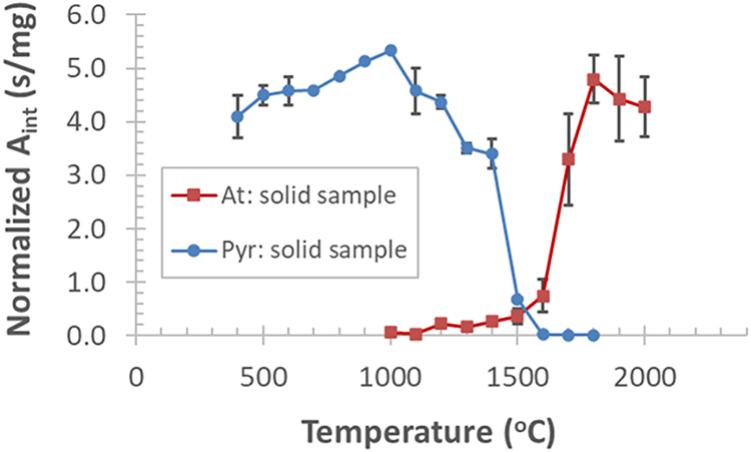
Pyrolysis and atomization curves of Bi recorded by dosing solid (powder) samples of a Bi-doped lithium niobate crystal with a Bi content of ≈190 μg/g; each data point and error bar represent mean ± SD, *n* = 3.

The pyrolysis curve slightly increases from 400 °C and starts to decline above ≈1000 °C. The atomization curve increases from ≈1100 °C, i.e., the appearance temperature (*T*_app_) of Bi. Interestingly, these data point to early vaporization of a small fraction of Bi from the LiNbO_3_ powder in the graphite furnace, below its melting point (mp) ≈1250 °C.^49^ As expected, the vaporization and atomization of Bi becomes faster above the mp of the host lattice, i.e., *T*_at_ > 1300 °C. Based on these data, the optimal *T*_pyr_ and *T*_at_ for solid LiNbO_3_ samples were found at 1000 and 1800 °C, respectively.

Solid sampling GFAAS methods usually rely on the use of solution standards, since solid standards of accurately known elemental composition are usually not available. Thus, the pyrolysis and atomization curves for solution standards of Bi acidified with HNO_3_ and/or added with TAC modifier were examined too (Figure 2). In HNO_3_ medium, the pyrolysis curve of Bi starts to decrease at ≈400 °C, though this decrease is slow until 900 °C, corresponding to the slow decomposition/low vaporization loss of Bi compounds at these temperatures. The addition of HNO_3_ (e.g., 0.1 mol/L, Figure S2) stabilizes Bi in the graphite furnace, likely in the form of Bi_2_O_3_ as explained below.

**Figure 2:**
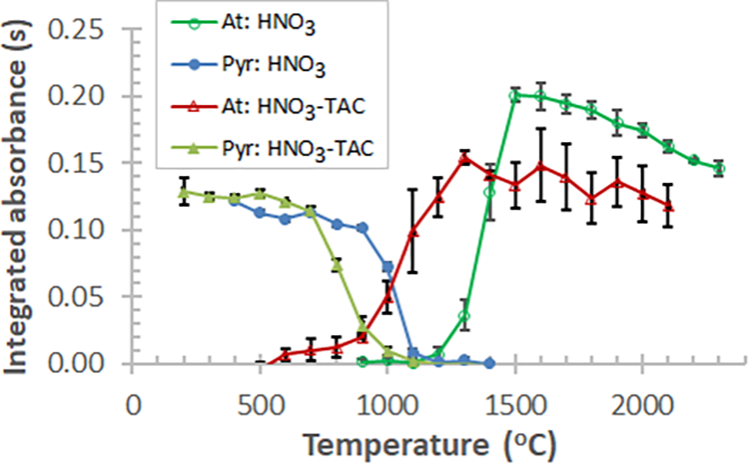
Pyrolysis and atomization curves for 20 μg/L Bi standards prepared with either 0.1 mol/L HNO_3_ or 0.1 mol/L HNO_3_ plus 0.05 mol/L TAC (premixed solutions); each data point and error bar represent mean ± SD, *n* = 3.

This stabilization effect is most likely encountered through an increase in the partial pressure of oxygen, which also blocks the active sites of graphite, thus preventing the reduction of Bi compounds at lower pyrolysis temperatures. The resulting effect can also be seen at the onset of the atomization curve, which appears at a higher temperature (*T*_app_ ≈1100 °C). In HNO_3_ medium, the optimal *T*_at_ is 1500 °C. On the other hand, in HNO_3_–TAC medium, the pyrolysis curve plateaus up to about 500 °C, whereas a slow absorbance decrease appears up to 700 °C, followed by a rather sharp decline. The *T*_app_ of Bi is lower (≈500 °C) compared to that observed with the HNO_3_ medium. In thermodynamic calculations, Frech et al.^50^ pointed out the chemical forms Bi_2_O_3_, Bi_2_, and Bi are present during the vaporization of bismuth compounds in graphite furnace atomizers in temperature ranges of 300–670, 730–930, and 670–1600 °C, respectively. Their results are consistent with the present findings, exemplifying the effects of oxidizing (HNO_3_) *versus* reductive (TAC) additives.

The solution sample GFAAS showed an entirely different pattern of pyrolysis and atomization curves compared to those observed for solid sampling (Figure 3). As appears, in modifier-free LiNbO_3_–HF–HNO_3_ medium, the pyrolysis curve possesses a plateau range up to 500 °C, whereas the addition of 0.05 mol/L TAC modifier only slightly increases the optimal *T*_pyr_, i.e., up to 600 °C. Interestingly, a significant difference in *T*_app_ of Bi can be seen for the same solution matrix without and with TAC, i.e., 500 and 800 °C, respectively. This is due to the halide removal effect of TAC modifier during the drying and/or low-temperature pyrolysis steps, which stabilizes Bi in the atomizer.

**Figure 3:**
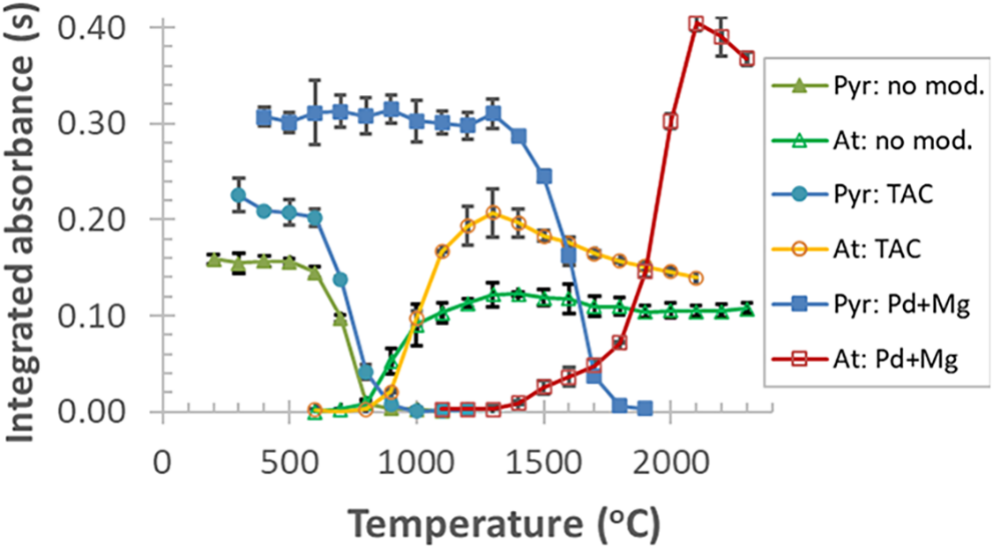
Pyrolysis and atomization curves of 20 μg/L Bi standards added with 0.01 mol/L LiNbO_3_ (undoped) in 0.8 mol/L HF–HNO_3_; modifier-free, and either with 0.05 mol/L TAC (premixed to standard) or 5 μg Pd + 1 μg Mg (applied as nitrates and dosed along with the standard); each data point and error bar represent mean ± SD, *n* = 3.

This effect may be encountered via sublimation of NH_4_F or melting (and vaporization) of NH_4_HF_2_ at 126 °C. Interestingly, for both types of sample solutions, the optimal *T*_at_ was observed at 1300 °C, suggesting that TAC does not considerably affect the atomization of Bi in the LiNbO_3_–HF–HNO_3_ medium. In contrast, when using the Pd–Mg modifier, a significant increase in the optimal *T*_pyr_ and *T*_at_ values was observed, i.e., to 1300 and 2100 °C, respectively. Ostensibly, the *T*_app_ is also much higher with the use of this modifier, i.e., ≈1400 °C. These findings point toward a firm action of the Pd modifier on Bi. Likely, the analyte forms a thermally stable compound in the graphite furnace during pyrolysis, such as intermetallic alloys of Pd,^44^ or even intercalation compounds within the surface of the graphite platform.^51^ Though MgO from the decomposition of Mg­(NO_3_)_2_ modifier can stabilize volatile Bi compounds in the furnace via the chemical pathway explained below. The effects encountered with the Pd–Mg modifier or the LiNbO_3_ matrix are in line with the observations of Volynsky^44^ and Tsalev et al.^52^ As an ultimate conclusion of the above results, the Pd–Mg modifier can be recommended for the solution introduction GFAAS analysis of LiNbO_3_ samples.

### 3.2 Effects of the Matrix and Chemical Modifiers

The effect of the matrix on the *A*_int_ signal of Bi was recorded using dissolved samples of undoped LiNbO_3_ over a wide concentration range, typical for solution GFAAS analysis (Figure 4, curve B). As seen, a smooth exponential increase in the Bi signal is observed starting from a matrix concentration of ≈0.002 mol/L, which peaks as high as 0.24 s at the largest matrix concentration applied. This result is somewhat unexpected, considering the usual decrease of the *A*_int_ signal for moderately volatile elements at higher LiNbO_3_ concentrations in the presence of halide-containing solvents.^9^ On the other hand, the action of increasing amounts of the LiNbO_3_ matrix on Bi can be explained by thermally stable Nb compounds formed in the graphite atomizer during pyrolysis.

**Figure 4:**
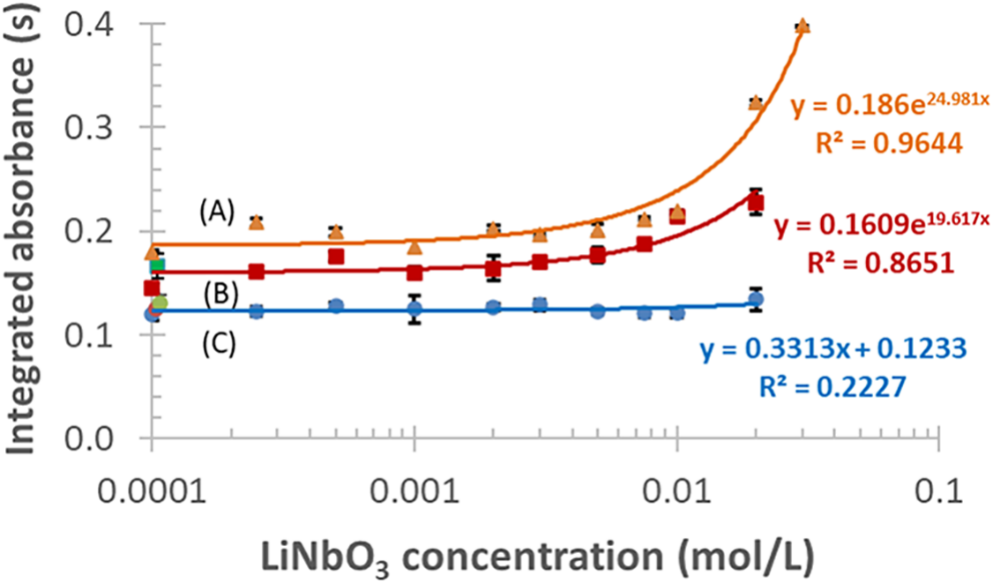
Effects of the dissolved LiNbO_3_ matrix on the *A*_int_ signal of 20 μg/L Bi; (A) with 5 μg of Pd + 1 μg of Mg (as nitrates), (B) without modifier, (C) with 0.05 mol/L TAC, (green data points correspond to matrix-free standards); each data point and error bar represent mean ± SD, *n* = 3.

This Nb-rich layer acts as an internal modifier, as it delays the vaporization of Bi during the atomization step, allowing the atomizer to reach a more isothermal state. This action eventually results in a longer absorption volume in the optical path. Moreover, it increases the residence time of the analyte atoms in the graphite furnace and consequently amplifies the absorbance signal. The uncertainty of the matrix-related measurements (Figure 4), expressed as RSD, ranged between 0.8 and 7.3% (average: 3.4%, median: 2.9%).

Interestingly, when the TAC modifier is used, the Bi signal remains nearly constant over the studied matrix concentration range (Figure 4, curve C). TAC has previously been applied to remove halide interference effects, i.e., the suppressed Cr signal, observed in the presence of LiNbO_3_.^9^ This provides a plausible explanation for the effect occurring in the current analysis, if one considers the action of TAC on the HF–HNO_3_ content of the sample solution, as mentioned above. On the other hand, the thermal stabilization mechanisms may be different, given the carbide-forming and reducing abilities of Bi compounds, as envisaged below.

When increasing the matrix concentration and applying Pd–Mg modifier (5 + 1 μg), the Bi signal remains constant up to 5 × 10^–3^ mol/L LiNbO_3_, but above this concentration it increases exponentially (Figure 4, curve A). This effect is due to a combined action of the matrix and the chemical modifier on the signal response of Bi. The RSDs of these determinations ranged from 0.7 to 5.4% (average: 3.0%, median: 2.7%). The higher *A*_int_ signal of Bi is a definite advantage of the Pd–Mg modifier over the TAC modifier or the matrix-matching method (see Section 3.4).

The increasing mass of TAC in matrix-free HNO_3_ medium causes a gradual increase of the Bi signal (Figure 5), even at a low TAC concentration of 1 × 10^–4^ mol/L, compared to a reference, TAC-free standard solution (Figure 5, red data point). At the highest TAC concentration applied, the signal reached around 1.4-fold of that observed for TAC-free solutions. The RSDs of these determinations increased from 2.8 to 11%, in proportion to the amount of TAC.

**Figure 5:**
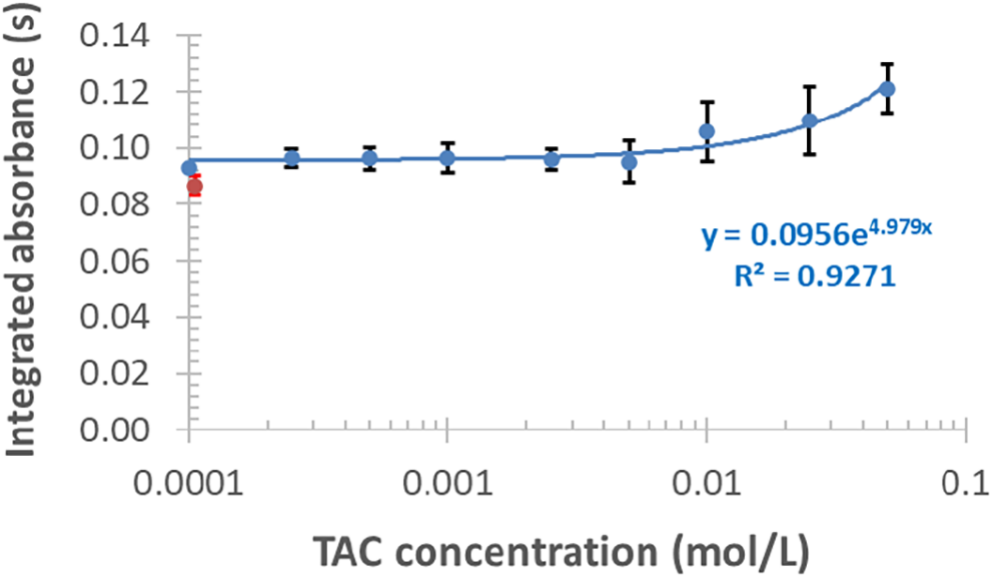
Effects of TAC modifier on the signal of 20 μg/L Bi (the red data point stands for a modifier-free standard); each data point and error bar show mean ± SD, *n* = 3.

The action of TAC in matrix-free standards appears to be obvious as follows. Solvents and ammonium salts vaporize during the drying/pyrolysis steps, e.g., as NH_4_NO_3_ (mp ≈170 °C, boiling point (bp) 210 °C), while the bismuth citrate salt remains in the graphite furnace as a pyrolysis residue of the sample. The decomposition of the latter compound is known in the thermoanalytical literature.^53^ In air, it happens via two steps at 250–325 and 360–400 °C, respectively, corresponding to disproportionation (intermediates: BiO, Bi_2_O_3_ (α- and β-phases), and BiO­(CH_3_COO)) and the formation of BiO and Bi_2_O_3_ (uptake of O_2_ from ambient air).^53^ The latter compound was reported to be stable up to 600 °C, while above this temperature, it takes oxygen from the ambient air. It should also be mentioned that the X-ray diffraction (XRD)/infrared (IR)-pattern of the original citrate compound disappears already at 300 °C. When decomposing bismuth citrate in a dry N_2_ atmosphere, one endothermic step at 250–325 °C leads to dissociation products, such as Bi and BiO.^53^ This mechanism is likely valid for graphite furnace atomizers operated in an argon protective atmosphere.

Based on these data, it is likely that the larger amount of citrate supplies excess oxygen in the gas/condensed phases during its decomposition in the pyrolysis step. Thus, it thermally stabilizes metallic Bi (mp 271 °C, bp 1560 °C), e.g., via conversion of bismuth citrates into the less volatile Bi_2_O_3_ (mp 825 °C, bp 1890 °C); thereby it vaporizes with delay in an atomizer at a higher and more homogeneous temperature. This causes a longer residence time of analyte atoms in the optical path, resulting in an augmented signal for Bi.

### 3.3 Atomization Transients, Vaporization of the Matrix, and Chemical Modifiers

The acidic (HNO_3_) standard of Bi in the presence of Pd–Mg modifier yields a symmetrical transient with an appearance time (*t*_app_) of 0.8 s (Figure 6a, blue curve). It peaks at 2 s with an aptly fast-falling edge, whereas dissolved samples of Bi-doped LiNbO_3_ with the same amount of Pd–Mg modifier show rather symmetrical and less prolonged transients (*t*_app_ ≈ 0.68 s), which peak at 2.1 s (Figure 6a, red curve). At higher Mg content (e.g., 2.5 μg) of the Pd–Mg modifier, broadened Bi signals for the doped crystal samples can be observed with multiple peaks (signal onsets at ≈1.3 s), while they reach their maximum at longer atomization time (3.0 s) (Figure S3). The transient signal of a blank recorded with undoped LiNbO_3_ and Pd–Mg modifier is depicted in Figure 6a (green dashed curve).

**Figure 6:**
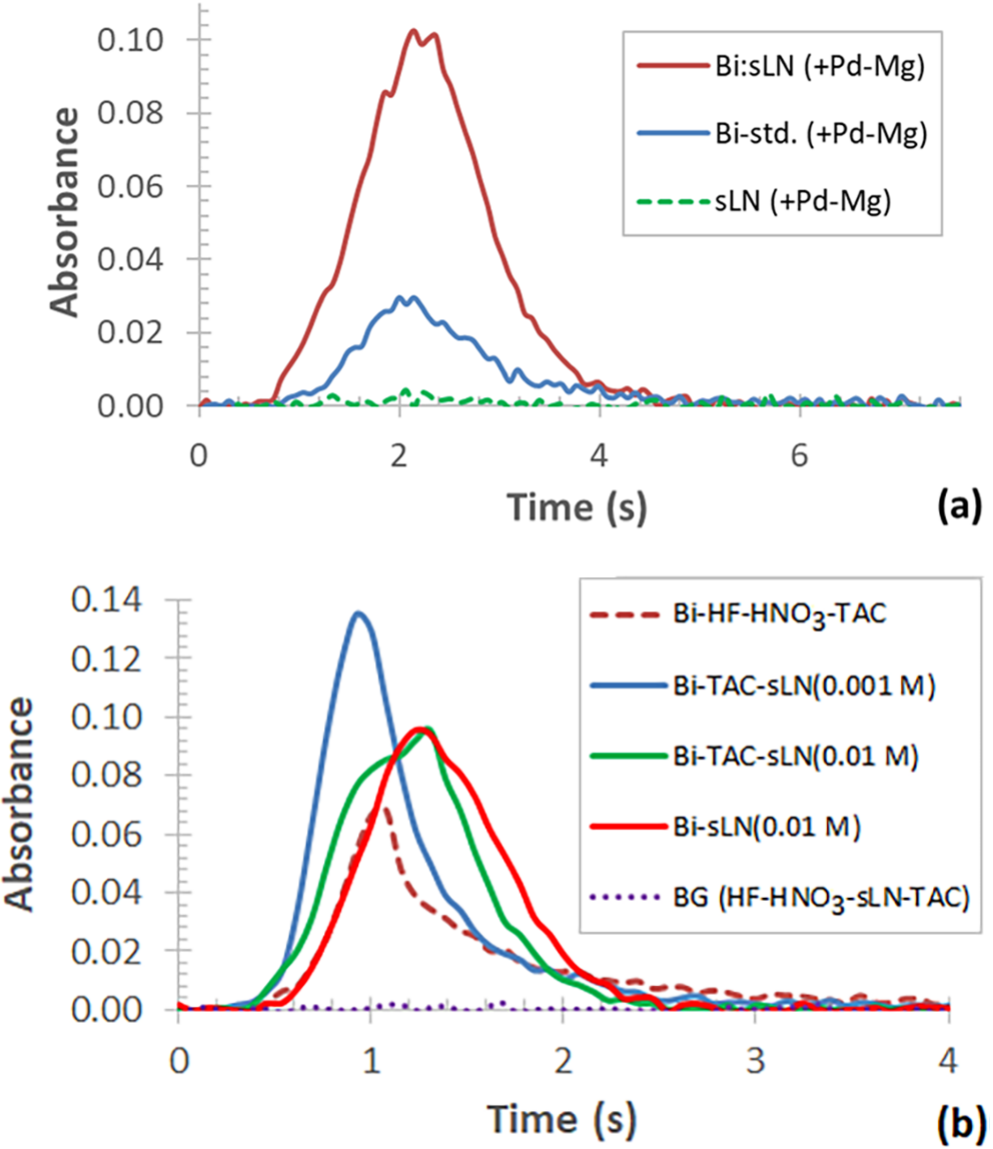
Atomization transients recorded with standard and sample solutions; graph (a): 5+1 μg Pd+Mg either with 20 μg/L Bi (blue curve), or 0.01 mol/L of Bi-doped LiNbO_3_ (red curve), or undoped LiNbO_3_ (green dashed curve); graph (b): 20 μg/L Bi either in 0.8 mol/L HF + HNO_3_ with 0.05 mol/L TAC (dark red dashed curve), or 0.001 mol/L undoped LiNbO_3_ and 0.05 mol/L TAC (blue curve), or 0.01 mol/L undoped LiNbO_3_ and 0.05 mol/L TAC (green curve); or modifier-free, 0.01 mol/L undoped LiNbO_3_ (light red curve); BG: blank for LiNbO_3_–HF–HNO_3_–TAC (purple dotted curve).

The atomization signal of Bi recorded with the digestion agent (HF + HNO_3_) and TAC modifier shows a rather sharp peak at 1.1 s (*t*_app_ ≈0.33 s), with an exponentially decaying edge, even at an atomization time as long as 4 s (Figure 6b, dark red dotted curve). This decay is due to the presence of TAC, which prolongs the vaporization of Bi in the graphite furnace, as explained above.

The atomization transient of Bi for dissolved samples of 1 × 10^–3^ mol/L LiNbO_3_ (HF + HNO_3_ media) with TAC can be described as a sharp, symmetrical signal (*t*_app_ = 0.2 s), peaking at ≈0.9 s (Figure 6b, blue curve). On the other hand, when one increases the concentration of LiNbO_3_ to 1 × 10^–2^ mol/L in the same medium, the transient broadens and becomes rather symmetrical, with a short delay (*t*_app_ ≈ 0.33 s) and a peak maximum at ≈1.3 s (Figure 6b, green curve). Interestingly, this signal shows a rather fast return to the baseline. When evaporating Bi in a modifier-free medium with the same amount of LiNbO_3_, a similarly broadened Bi transient was observed, but with a significantly delayed onset (*t*_app_ ≈0.5 s) and a peak maximum at 1.9 s (Figure 6b, red curve). This effect is due to the matrix, as it acts as an internal modifier by prolonging the vaporization of Bi.

When using standards acidified with HNO_3_, the transient signal of Bi appeared as a broadened peak with a slight exponential decay (*t*_app_ = 0.4 s), peaking at 0.9 s, likely showing delayed decomposition and vaporization of Bi_2_O_3_ in the graphite furnace (Figure 7, green curve). On the other hand, if this medium is used with the addition of TAC, the Bi transient shows greater symmetry with slightly shorter *t*_app_ and peak maximum, but a smaller delay at the trailing edge (Figure 7, red curve).

**Figure 7:**
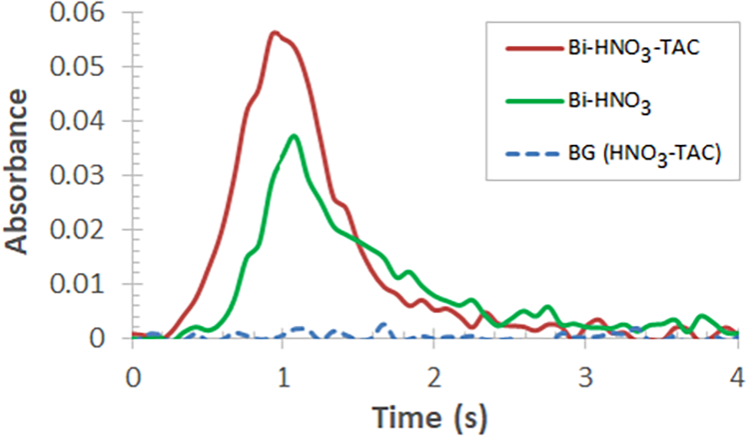
Atomization transients of 20 μg/L Bi in 0.1 mol/L HNO_3_; modifier-free (green curve) or with addition of 0.05 mol/L TAC, premixed (red curve); background (BG) absorbance for 0.1 mol/L HNO_3_ + 0.05 mol/L TAC (blue curve).

When analyzing solid (powder) samples of LiNbO_3_, a spike-shaped atomization transient of Bi could be seen with long *t*_app_ (3.5 s) and a peak maximum at 3.9 s (Figure 8a, red curve). When a 1.7-times greater mass of the same sample was dispensed, a broadened Bi signal was observed with an onset of ≈1.25 s and double peaks at 2.8 and 4.1 s (Figure 8a, blue curve). This effect is likely due to the release of Bi from a powder of non-homogeneous size distribution, resulting in early-stage decomposition and vaporization of the finer fraction. However, upon addition of 5 μL of acidic (HNO_3_) standard, Bi vaporizes earlier in the atomization step, at *t*_app_ of ≈0.9 s and reaches its peak at ≈1.8 s for the solution standard, while the peak for the solid sample appears later (4.4 s) (Figure 8a, green dotted curve), compared to the case, when it is dispensed alone into the graphite boat (e.g., Figure 8a, red curve). A similar pattern was observed for other Bi transients when the volume of the standard solution was further increased (Figure 8b).

**Figure 8:**
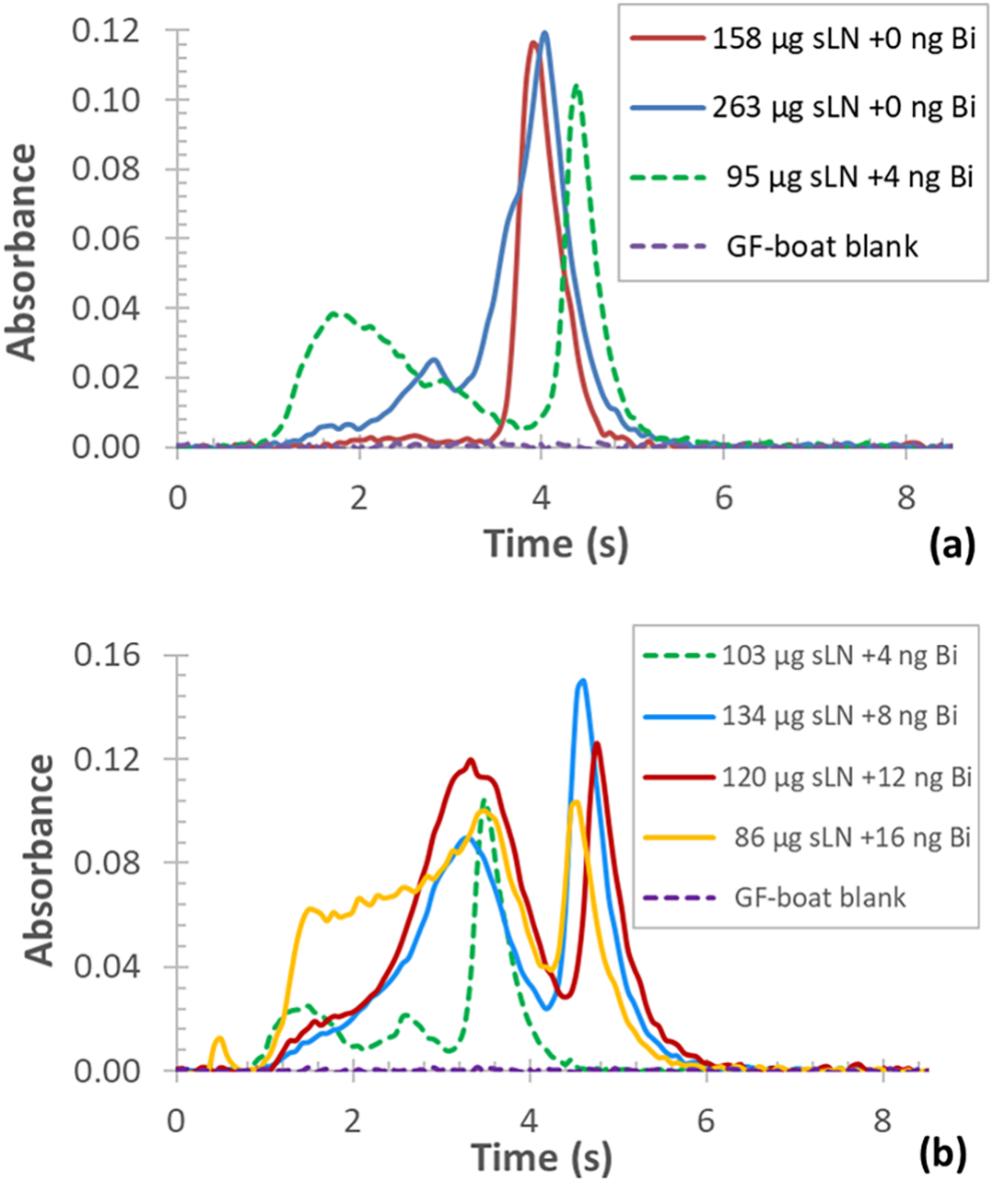
Absorbance *vs* time curves of Bi for solid (powder) samples of LiNbO_3_ without (a) and with calibration standards (b) of varying volumes (dispensed mass of Bi is indicated).

Interestingly, when 20 μL of the standard (equal to 16 ng of Bi) was added to the solid sample, the Bi signal broadened considerably. This effect, besides the early-stage vaporization and atomization of Bi (*t*_app_ ≈ 0.35, ≈0.9 s), was manifested in the appearance of multiple peaks at 0.53, 1.6, 3.5, and 4.5 s (Figure 8b, amber curve).

To explain the double peaks appearing in the atomization transients of Bi, Takada and Hirokawa^54^ proposed the evaporation of Bi from differently bound forms of alloy (solid) samples, i.e., from near grain boundaries and surfaces, while the twin-peak was attributed to the crystal bulk of the sample. The current findings suggest similar mechanisms for the double peaks observed for Bi-doped LiNbO_3_ powder samples dosed without standard solutions. Namely, low Bi peaks were observed in the early stage of atomization, representing a small fraction of the analyte mass (up to ≈10%), whereas the larger amount of the analyte vaporizes at a later stage of atomization, corresponding to a higher atomizer temperature and undoubtedly the decomposition of crystalline LiNbO_3_ powder.

For the LiNbO_3_-related studies, two-dimensional (2D) transients were sufficient to depict the temporal evolution of the atomization signal, due to the simple spectral proximity of the analytical lines of Bi. However, for the CRM (laterite soil), three-dimensional (3D) transients were evaluated due to the complex chemical space of this matrix (Figure 9). As can be seen, the Bi I 223.0608 nm analytical line appears in the middle of the spectral window with a relatively short decay (*t*_app_ ≈ 1.6 s) and a peak maximum at 2.55 s. In the vicinity of the Bi line, transients of complex band systems of molecular species (SiO, AlH) can be seen, formed from covaporization of the matrix. These bands experience a significant delay (*t*_app_ ≈3.8 s) compared to the Bi transient. The observed bands correspond to the X^1^Σ^+^ → A^1^Π and the X^1^Σ^+^ → C^1^Σ^+^ electronic transitions of the SiO 223.63 nm and the AlH 222.86 nm band systems, respectively.^47^ It is to be noted that these bands and the Bi line cannot be resolved, nor can the arising structured background be compensated using LS-GFAAS, but HR-CS-GFAAS.

**Figure 9:**
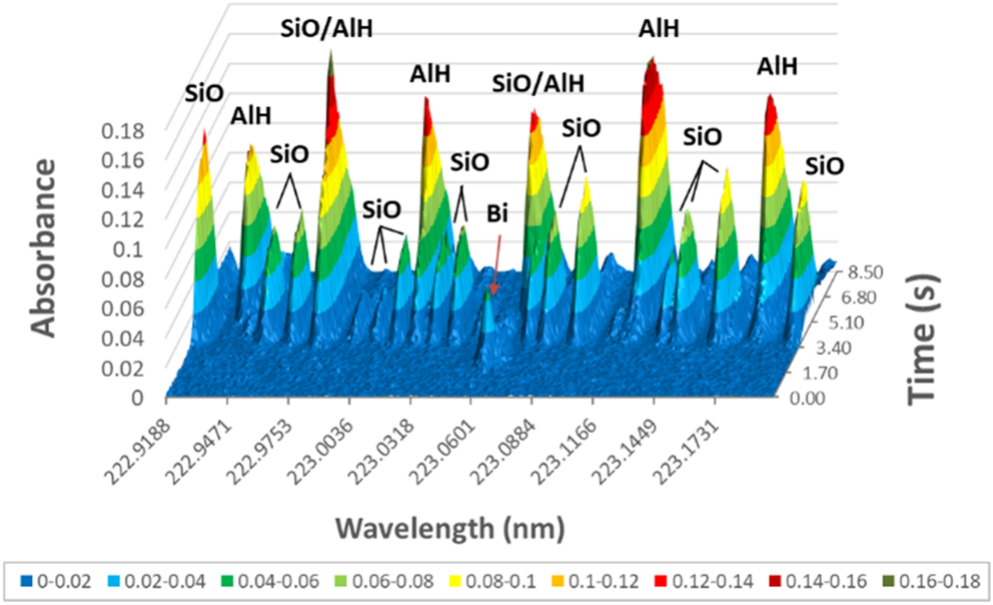
Spectrally resolved absorbance–time curves for 63 μg powder sample of the CRM (GBW07407) in the vicinity of the Bi I 223.0608 nm analytical line.

Overall, based on the observed signals of the sample matrix, whether dispensed in solution or in solid (powder) form, it becomes necessary to prolong the atomization and signal integration time in the furnace heating program, compared to those observed for acidic calibration standards.

### 3.4 Calibration Methods and Analytical Performance

For the solution sample GFAAS, the slopes and intercepts of the calibration equations determined using acidic standards, matrix-matching, and/or the addition of chemical modifiers (Pd–Mg or TAC) were slightly different (Table 2). However, similar LOD values were obtained for these methods (≈1.2 μg/L), corresponding to an LOD of ≈0.8 μg Bi/g crystal sample. An exception was found for the calibration aided by the Pd–Mg modifier, providing an improved LOD of 0.4 μg/L, corresponding to ≈0.27 μg Bi/g crystal. Consequently, this method was selected for the solution analysis of LiNbO_3_ samples.

**Table 2:** Calibration and Analytical Performance for the Developed Solid and Solution Sample GFAAS Methods

calib. method	calibration equation^a^	LOD (μg/L)	LOD (μg/g)	LOQ (μg/g)	*m*_o_ (pg)
acidic std.	*A*_int_ = 0.0044*c*_Bi_ + 0.0241	1.2	0.80^b^	2.7	17.4
matrix-matching	*A*_int_ = 0.0046*c*_Bi_ + 0.0095	1.1	0.83^b^	2.8	16.6
Pd–Mg modifier	*A*_int_ = 0.0061*c*_Bi_ + 0.0003	0.4	0.27^b^	0.9	14.6
TAC modifier	*A*_int_ = 0.0043*c*_Bi_ + 0.0089	1.3	0.90^b^	3.0	17.7
convent. std. add.^c^	*A*_int‑n_ = 0.196*c*_Bi_ + 0.942	n.a.	0.45^d^	1.5	220
three-point std. add.^c^	*A*_int_ = 0.0175*c*_Bi_ + 0.0961	n.a.	0.38^d^	1.3	226

a *c*_Bi_ — Bi concentration.

b Sample mass: ≈0.07 g (analytical line: Bi I 223.0608 nm).

c For solid sample analysis.

d Average sample mass: ≈0.1 mg (analytical line: Bi I 227.6580 nm), *A*_int‑n_ — integrated absorbance normalized to sample mass. n.a. — not applicable.

The LOD values of the current solution sampling GFAAS methods are more or less commensurate with the literature data, which range from 0.001 to 8.5 μg/L (Table 3). This wide scale of LODs is due to the analyte preconcentration/matrix separation procedures used in some of these methods prior to GFAAS analysis. Application of these procedures was certainly not the primary aim of the present study, but rather to achieve a suitable Bi signal response from the samples.

**Table 3:** Analytical Performance of the Current Solution GFAAS Method and Literature Methods for Bi^a^

method	matrix/sample	modifier	LOD (μg/L)	*m*_o_ (pg)	ref
digestion in HNO_3_ (HClO_4_–HF), DEDTP-complex, FIA-preconc. in knotted PTFE	cod muscle, lake- and river sediment	-	0.003	-	^14^
digestion in HNO_3_–HClO_4_–HF mixture	geological, seawater	Ni–Pd–TA, Ni–Pt–TA	6.5, 6.9	163, 180	^15^
digestion in HNO_3_–HClO_4_	geological	W	-	-	^16^
acid addition	spiked seawater	W–Pd–TA	8.5	70	^17^
direct analysis, acid addition	artificial and real seawater	Mo–Pd–TA	7.0	51.6	^18^
WF-AAS electrochem. separ. in Mg–W-cell	environmental	-	0.0078	-	^19^
solvent extraction	urine, sea- and wastewater	Pd	0.02	-	^20^
SPE, octadecyl-bonded silica cartridge modified with Cyanex-301	water, alloy	-	0.01	-	^21^
SPE-preconc., silica gel modified with 3-amino-propyltriethoxysilane	seawater	-	0.5	-	^22^
SPE on Chromosorb-107, eluted with HNO_3_	CRMs: apple leaf, waste- and seawater	-	0.8	-	^23^
FIA-preconc. MW-dig. stannite precip./collect. on knotted coil reactor	biological	-	0.04	12	^25^
MW-digestion complexation-preconc. on activated carbon, elution in low vol. HNO_3_	steel	Pd–Mg	≈0.02	-	^26^
preconc. on activated carbon, elution in ethanol	steel	permanent Ir	0.048	-	^27^
complexation, FIA sorption-preconc. on activated carbon	CRM steel, Al-alloy (foil)	permanent Ir	0.25	-	^28^
preconc. magnetic core–shell silica NPs modified with AAAPTS	human hair, tap- and well water	-	0.0014	-	^30^
FIA-HG-GF-in-atomizer-trapping	water	permanent Ir	0.13	55	^55^
preconc. on XAD-1180	high-purity Al, Zn, steel alloy	-	4.0	-	^56^
cloud point extraction	biological, tap water	Pd(NO_3_)_2_	0.02 (1.5)	-	^57^
liquid–solid microextraction	human hair and blood serum	Pd + NH_4_H_2_PO_4_	0.16	-	^58^
Ag-NP-preconc. pistachio skin extract.	water, food, human serum and hair	-	0.09	-	^59^
FIA-SPE-CVG, funct. silica, HR-CS-GFAAS	fortified lake water CRM	permanent Ir	0.001	-	^60^
MW-digestion in acids	LiNbO_3_	Pd–Mg	0.4	15	this study

a Abbreviations: LOD—limit of detection, m_o_—characteristic mass, FIA—flow injection analysis, TA—tartaric acid, funct.—functionalized, MW—microwave, NP—nanoparticle, SPE—solid phase extraction, CRM—certified reference material, WF—tungsten furnace, DEDTP—diethyldithiophosphate, AAAPTS—3-[2-(2-aminoethylamino)­ethylamino]­propyl-trimethoxysilane, HG—hydride generation, CVG—cold vapor generation.

The characteristic mass (*m*_o_), by definition, is the amount of the analyte, yielding an *A*_int_ signal of 0.0044 s.^61,62^ This parameter is generally applied in GFAAS to give the absolute sensitivity of the method. In this study, *m*_o_ values of various calibration methods (Table 2) range from 14.6 to 17.7 pg with an average of 16.6 ± 1.4 pg (median: 17 pg), which is in good agreement with that specified by the manufacturer of the AAS instrument (17 pg). Only a couple of *m*_o_ values for Bi have been published in the surveyed literature (Table 3), covering a wide range from 12 to 180 pg (average: 89 pg; median: 63 pg). This variation of the reported methods in sensitivity is likely due to the use of different analytical technologies and furnace designs, based mostly on conventional LS-GFAAS.

Sample mass in GFAAS has been reported to influence analytical accuracy.^63^ Too large a sample mass can result in underestimation of the analyte concentration, whereas too little sample amount can lead to its overestimation.^63^ Consequently, the linearity of the *A*_int_ signal response *vs* sample mass curves were examined for a couple of LiNbO_3_ powder samples (Supporting Information, Figure S4a–k). As appears, the curves are linear over a broad sample mass range (30–300 μg) and can be characterized by slopes of 0.68–4.46 s/mg, corresponding to crystal samples with different Bi dopant concentrations.

As a first approach to solid sample analysis, conventional standard addition calibration was applied to the powdered LiNbO_3_ samples (Figure 10a). As appears, a linear relationship was established for the calibration points.

**Figure 10:**
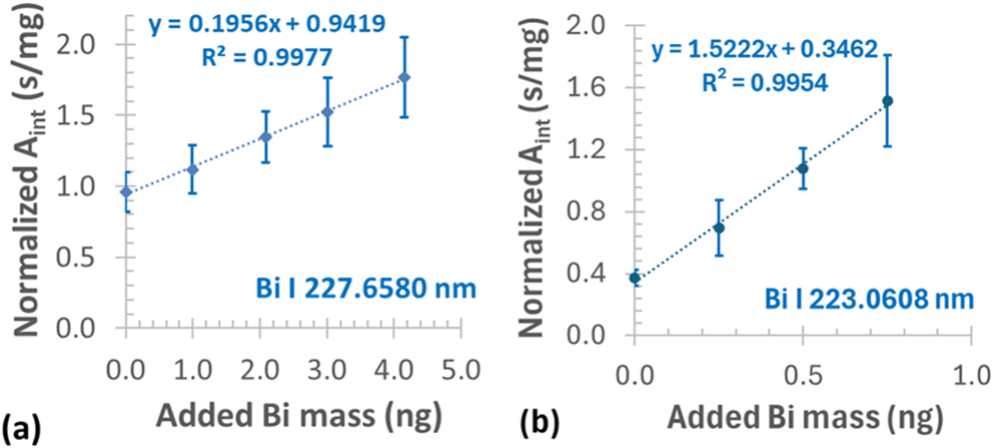
Conventional standard addition calibration for the solid sample GFAAS determination of Bi in a doped LiNbO_3_ crystal (a), and in the GBW07407 soil CRM (b); recorded with acidic (0.015 M HNO_3_) standards of 0.2 mg/L Bi (a) and 0.05 mg/L Bi (b); each data point and error bar represent mean ± SD, *n* = 10–15.

The accuracy of the solid sampling method was verified using the CRM GBW07407 (laterite soil) with a certified Bi content of 0.20 ± 0.04 μg/g. This analysis was performed at the more sensitive Bi I 223.0608 nm line, due to the low Bi content of the CRM, but using a higher atomization temperature of 2300 °C to ensure complete vaporization of the sample. The conventional standard addition calibration performed with the CRM sample gave a Bi content of 0.227 ± 0.031 μg/g, which corresponds to a recovery of 113%. A typical standard addition calibration recorded with the CRM sample is depicted in Figure 10b.

As the next approach, the three-point estimation standard addition was applied to calibrate the solid sample analysis of LiNbO_3_. This procedure is already well-documented^64^ and applied in the AAS literature.^9,64^ As appears in Figure 11, an increase in the added Bi calibration standard proportionally enhanced the intercepts of the calibration curves, corresponding to the desired linear increment. While the slopes of the calibration equations approximated a similar value (0.66–0.68). The correlation coefficients (*R*) of the curves ranged from 0.90 to 0.98, pointing to a strong correlation of *A*_int_ *vs* sample mass. From the slope and intercept pairs of each equation, the absorbance values of the three-point calibration curve were calculated for the average dispensed sample weight of ≈0.1 mg, applied to the current solid sampling GFAAS analysis.

**Figure 11:**
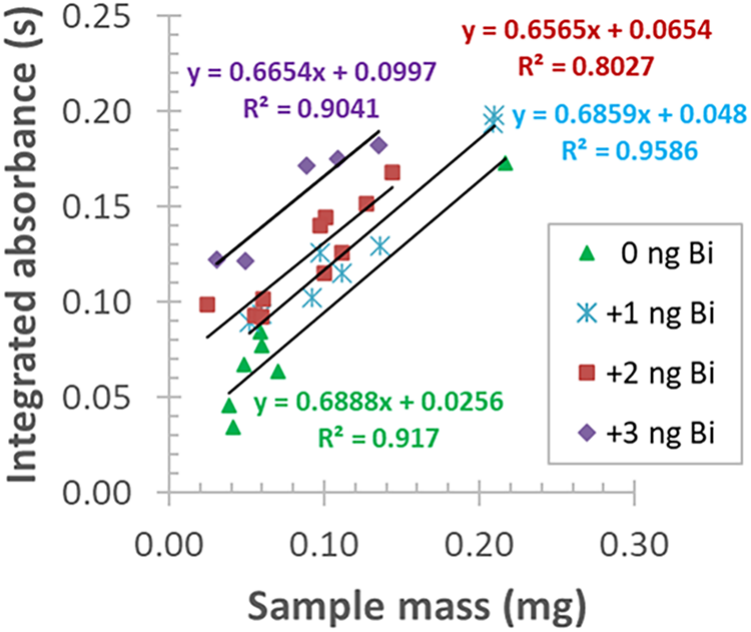
Three-point estimation standard addition calibration with increasing volumes of an acidic standard (0.2 mg/L Bi) for the determination of Bi at 227.6580 nm in a doped LiNbO_3_ sample.

The LOD was found to be 0.45 and 0.38 μg/g for solid sampling GFAAS with conventional standard addition and the three-point estimation method, respectively, using a less sensitive Bi line at 227.6580 nm. This line has 7% of the sensitivity compared to the most sensitive resonant line of Bi at 223.0608 nm. Applying the latter, an order of magnitude better LOD can be achieved, i.e., 0.03 μg/g.

Regarding the solid sampling GFAAS literature, Bäckman and Karlsson^65^ determined Bi in steel and nickel-based alloys and achieved an LOD as low as 0.03 μg/g, when dosing sample amounts of 1–20 mg. Hiltenkamp and Jackwerth^66^ quantified traces of Bi in gallium by GFAAS using a sample mass of 5 mg, resulting in an LOD in the 0.1 μg/g range. Electrolytically spiked samples of high-purity gallium were used for calibration. Two orders of magnitude improvement in LOD were observed, compared to the solution GFAAS method, which uses larger amounts of partially dissolved samples. Terashima et al.^24^ developed a GFAAS method for the determination of Bi in a great variety of geological CRMs and reached an LOD as low as 0.8 ng/g, but applying an exceptionally high sample mass of 0.5 g. Dobrowolski et al.^37^ used slurry sampling GFAAS and found an LOD of 0.05 μg/g and a *m*_o_ of 16 pg for Bi, whereas Hinds et al.^67^ applied a solid sampling electrothermal vaporization-inductively coupled plasma-mass spectrometry (ETV-ICP-MS) method to quantify Bi in Ni-alloys by covaporizing seawater as a sample aerosol carrier and utilizing external calibration. By inserting samples up to 3 mg, an LOD of 0.014 μg/g was obtained in reduced sensitivity mode, whereas an LOD of 0.002 μg/g was acquired in high sensitivity mode. Schäffer and Krivan^68^ reported a solid sampling ETV-ICP-OES method for the determination of impurities in graphite and silicon carbide and obtained an LOD as low as 0.04 μg/g for Bi. Headridge and Riddington^69^ determined the Bi content in standard steel and Ni-base alloys using graphite microboats in induction- or resistance-heated GFAAS atomizers, and performing calibration against metallic standards and aqueous solution standards. Using a similar methodology in a later work,^70^ they quantified Bi in glass samples and achieved an LOD of 0.02 μg/g. A close LOD value was reported by Marks et al.^71^ for the GFAAS determination of Bi in complex, nickel-based alloy chips, when utilizing different designs of end-heated graphite atomizers (HGA, CRA) and cast alloy samples for calibration. Takada and Shoji^72^ quantified Bi in high-purity tin samples (5–25 mg) by GFAAS, and reported an LOD of 0.01 μg/g. As can be seen from this survey, the literature LOD data for GFAAS and ETV-ICP-OES are generally comparable to those values observed with the current solid sampling HR-CS-GFAAS method. On the other hand, one should notice the difference between the applied sample masses reported in the GFAAS literature (generally 1–500 mg) and those applied in the present study (on average ≈0.1 mg).

The literature has already reported data on experimental *m*_o_ values for solid sampling GFAAS methods, e.g.,^73,74^ but have not yet seen a formula for the same. Here, we suggest a modified version of the well-known equation for solution sample GFAAS analysis,^75^ and calculate the experimental *m*_o,solid_ (in pg) for the current solid sampling GFAAS method, involving the applied solution standard addition calibration procedure as follows:

1 $$m_{o,\mathrm{solid}}=\frac{0.0044\cdot v\cdot c}{{(A}_{i,n1}-A_{i,n2})\cdot m_{a}-A_{i,\mathrm{bl}}}$$

where *v* is the volume (in μL) of the calibration solution dispensed, *c* is the concentration of the standard solution (in μg/L), *A*_i,bl_ is the average absorbance for acidic blanks (in s), dispensed into the graphite sample boat, *A*_i,n1_ and *A*_i,n2_ are the average values of the normalized integrated absorbance for two of the calibration points, e.g., with and without adding the standard solution (s/mg), while *m*_a_ is the average mass of the sample (in mg), dispensed into the graphite atomizer without the use of calibration standards.

This formula yields *m*_o,solid_ of 220 and 226 pg for the conventional and three-point estimation standard addition methods, respectively. This data is consistent with the expected value of the current solid sampling GFAAS methods. When these data are compared with those obtained by solution GFAAS analysis (Table 2), the sensitivity ratio of the Bi lines used for solution and solid sampling is 14.3, which agrees with the ratio of the characteristic masses.

### 3.5 Analytical Results and Incorporation of Bi into the Host Crystal

The Bi dopant concentrations applied in the crystal growth melt and the analytical results observed for solid and solution sample GFAAS are listed in Table 4. The Bi concentration in the doped crystals obtained by solid sampling ranged between 55 and 311 μg/g, while it was lying between 56 and 296 μg/g for solution GFAAS. According to the *t*-tests, good agreement was found between the results obtained with the solid and solution sample introduction methods. The bias between the solution introduction and solid sampling three-point estimation standard addition methods of GFAAS ranged from 1.2 to 10% (average: 4.1%), whereas it was a bit higher between conventional standard addition and solution-based methods (range: 0.3–21%, average: 12%). The precision of solid and solution sample GFAAS methods was not worse than 6–16% (average: 12%) and 1.2–13% (average: 4.4%), respectively.

**Table 4:** Analytical Results for Solid Sampling and Solution Introduction GFAAS Methods

		average Bi content (±SD) in the crystal (μg/g)		
crystal No.^a^	initial Bi (mol %)^b^	solid (three-point estim. std. add.)	solid (convent. std. add.)	solution analysis
1	0.0	0.37 ± 0.14^c^	0.44 ± 0.17^c^	nd
2	0.5	54.9 ± 9.4	56.5 ± 9.3	56.7 ± 7.3
3	0.5	67.2 ± 6.5	74.2 ± 10.2	61.1 ± 1.8
4	2.0	185 ± 17	213 ± 15	192 ± 8
5	2.0	143 ± 20	155 ± 18	141 ± 2
6	4.0	277 ± 44	312 ± 28	296 ± 5
7	4.0	190 ± 22	196 ± 25	184 ± 9
8	2.0	134 ± 13	155 ± 13	137 ± 2
9	2.0	92 ± 6	106 ± 7	89 ± 2
10	4.0	241 ± 37	261 ± 59	233 ± 23
11	4.0	182 ± 22	201 ± 22	174 ± 4

a Crystal Nos. 8–11 are codoped with 1–2% Mg.

b Bi concentration applied in the starting material (crystal growth melt); SD—standard deviation (*n* = 3); nd—not detected (<LOD).

c Near LOD values.

The extent of Bi incorporation into the host crystal at different concentrations during growth can be assessed by plotting the initial (added) and observed Bi concentrations in the crystal bulk and performing various fits to the data sets (Figure 12). For crystals grown with the Czochralski method, the concentration pairs follow a logarithmically declining function with a high correlation coefficient (*R* = 0.9958). It can be seen that the incorporation efficiency of Bi into LiNbO_3_ is low, ranging from 0.3 to 0.9% of the added Bi amount. This function also shows that increasing the dopant concentration in the crystal growth melt results in lower incorporation; i.e., the segregation coefficient of Bi gradually decreases. On the other hand, for crystals grown by the HTTSG method, a larger decrease in the effective segregation coefficient was observed with increasing dopant concentration (Figure 12, red curve). But during this type of crystal growth process, the Bi dopant, applied as Bi_2_O_3_, competes with the flux additive (K_2_O) for incorporation into the host lattice.

**Figure 12:**
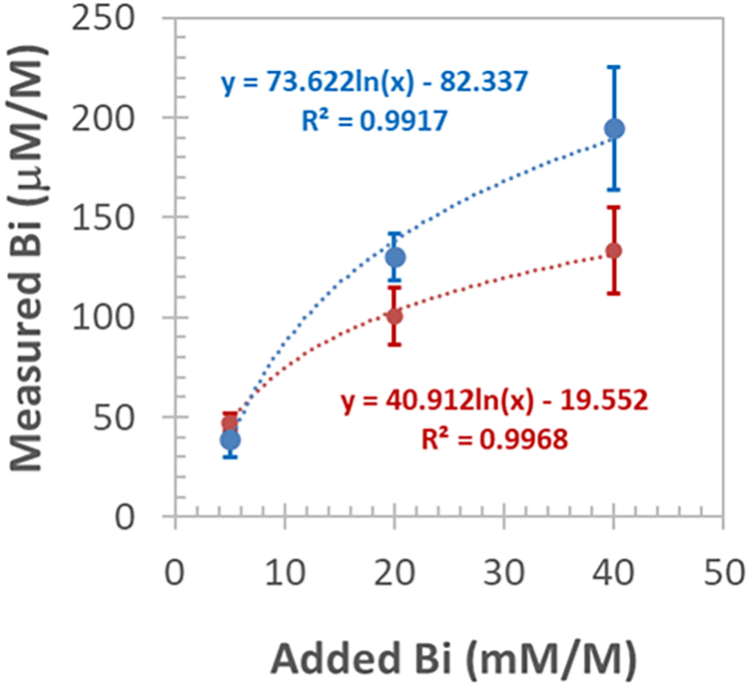
Dopant concentration in the crystal growth melt *vs* that found in the crystal samples by means of GFAAS; Bi-doped LiNbO_3_ crystals grown with the Czochralski method (blue curve), or the HTTSG method (red curve); each data point and error bar represent mean ± SD, *n* = 3.

## 4 Conclusions

In this study, HR-CS-GFAAS methods were elaborated for the determination of Bi in optical crystals of LiNbO_3_. Two types of solid sampling methods and some solution introduction methods for calibration were compared. Statistical tests showed good agreement between the analytical data obtained with solid and solution sample GFAAS. The advantage of the solid sampling method is the low sample mass demand (0.1 mg on average) and that the LOD can be extended to as low as 0.03 μg/g, using the resonance line of Bi I 223.0608 nm, instead of the less sensitive line (Bi I 227.6580 nm) utilized in this study for practical reasons. The solid sampling method was also tested on a soil CRM with a Bi content much lower than that of Bi-doped lithium niobate crystals. Moreover, an easy formula was suggested to calculate the experimental characteristic mass of the solid sampling GFAAS method.

No halide interference was observed in the presence of the mixed acid used as the dissolution reagent for the crystals. On the other hand, the organic salt modifier (TAC), when applied in excess, counteracted the positive effect of LiNbO_3_ matrix over a wide concentration range. Although the TAC modifier applied in increasing amounts caused gradually increasing and higher RSDs of the determinations, the Pd–Mg modifier was proven to be the best choice for solution sample analysis. In the case of the LiNbO_3_ matrix, the HR-CS-GFAAS method lacks spectral overlaps with the analytical lines. Thus, it is likely that this method can be adapted to conventional LS-GFAAS instruments. However, it is important to use THGAs to reduce memory effects that can occur during the analysis of the concerned refractory matrices. On the other hand, the analysis of the CRM must be performed with HR-CS-GFAAS, due to the complex spectral vicinity of the analytical lines utilized for the quantitation of Bi.

For solution samples, the LiNbO_3_ matrix showed a high retention/trapping efficacy on Bi in terms of stabilizing the analyte at higher pyrolysis temperatures in the graphite atomizer. This effect can be utilized for general Bi determination in other types of samples (e.g., ore, alloy, water), likely by applying only the refractory main component (Nb) of the crystal as a modifier. It should be noted that the currently developed methods are relevant in ETV-coupled techniques, such as ICP-OES and ICP-MS, due to their easy adaptability.

## Supplementary Material



## Notes

Effect of increasing mass of the Pd–Mg modifier on the absorbance signal of Bi (Figure S1); atomization–time curves recorded for Bi in the presence of increasing concentrations of HNO_3_ (Figure S2); atomization transients for solution samples of undoped and Bi-doped LiNbO_3_ with various amounts of Pd–Mg modifier (Figure S3); and linear fits to integrated absorbance of Bi (at 227.6580 nm) *vs* sample mass data for doped and undoped stoichiometric LiNbO_3_ (sLN) samples (Figure S4a–k) (PDF)
