# Supplementary material for: High-Resolution Graphite Furnace Atomic Absorption Spectrometry Determination of Bismuth in Lithium Niobate Optical Crystals
Source: ACS Omega. 2025 Oct 5;10(40):47082–94. doi: 10.1021/acsomega.5c05486 (PMC12529150; doi:10.1021/acsomega.5c05486)

High-Resolution Graphite Furnace Atomic Absorption Spectrometry Determination of Bismuth in Lithium Niobate Optical Crystals

Dániel Csontos[1], László Kovács[1], Krisztián Lengyel[1], and László Bencs[1,*]

[1] Department of Applied and Nonlinear Optics, Institute for Solid State Physics and Optics, HUN-REN Wigner Research Centre for Physics, P.O. Box 49, H-1525 Budapest, Hungary

* Corresponding author, e-mail: bencs.laszlo@wigner.hun-ren.hu

Figure S1. Signal response of 20 µg/L Bi (at Bi I 223.0608 nm) to increasing amounts of Pd–Mg chemical modifier, applied as nitrate solution at an optimal 5:1 ratio (data points represent blank corrected averages, while error bars indicate standard deviations).

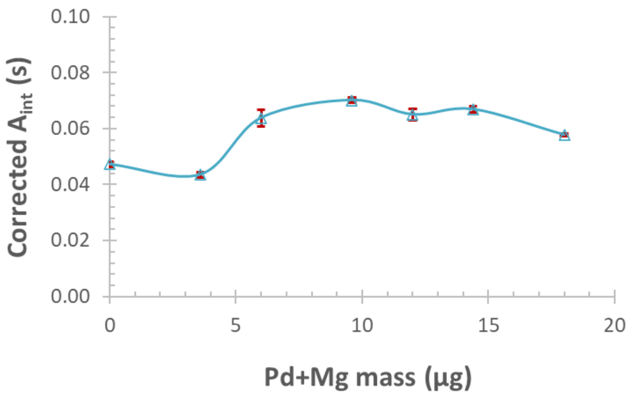

Figure S2. Atomization–time curves recorded at Bi I 223.0608 nm for standard solutions of 20 µg/L Bi, at increasing HNO_3 concentrations of 0.01, 0.1 and 1.0 mol/L with integrated absorbance values of 0.0218, 0.0311, and 0.0367 s, respectively.

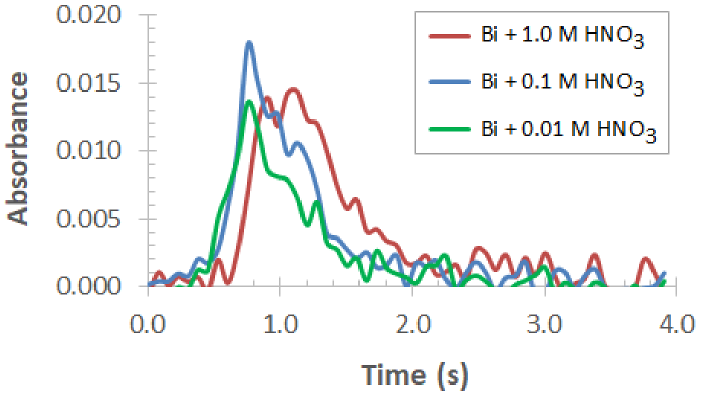

Figure S3. Atomization transients for solution samples of undoped and Bi-doped $LiNbO_3$ with various amounts of Pd+Mg modifier (5+2.5 µg, or 5+1 µg, respectively); crystal Nos. as in Table 4.

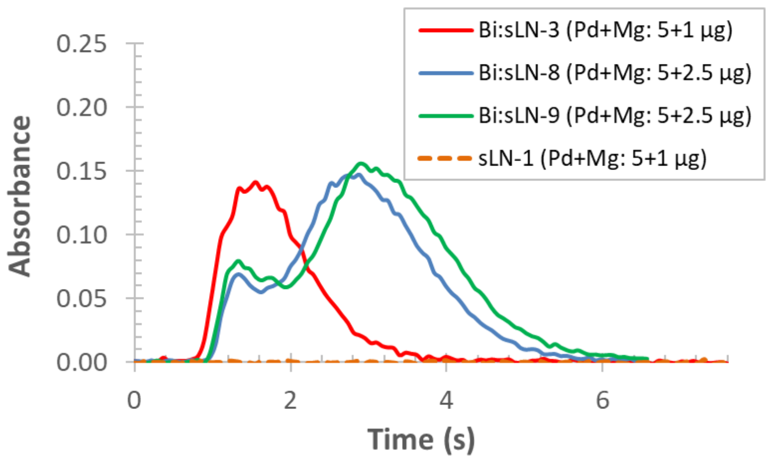

Figure S4. Linear fits to integrated absorbance of Bi (at 227.6580 nm) *vs.* sample mass data for doped and undoped stoichiometric $LiNbO_3$ (sLN) samples (crystal IDs are listed in Table 4 of the article).

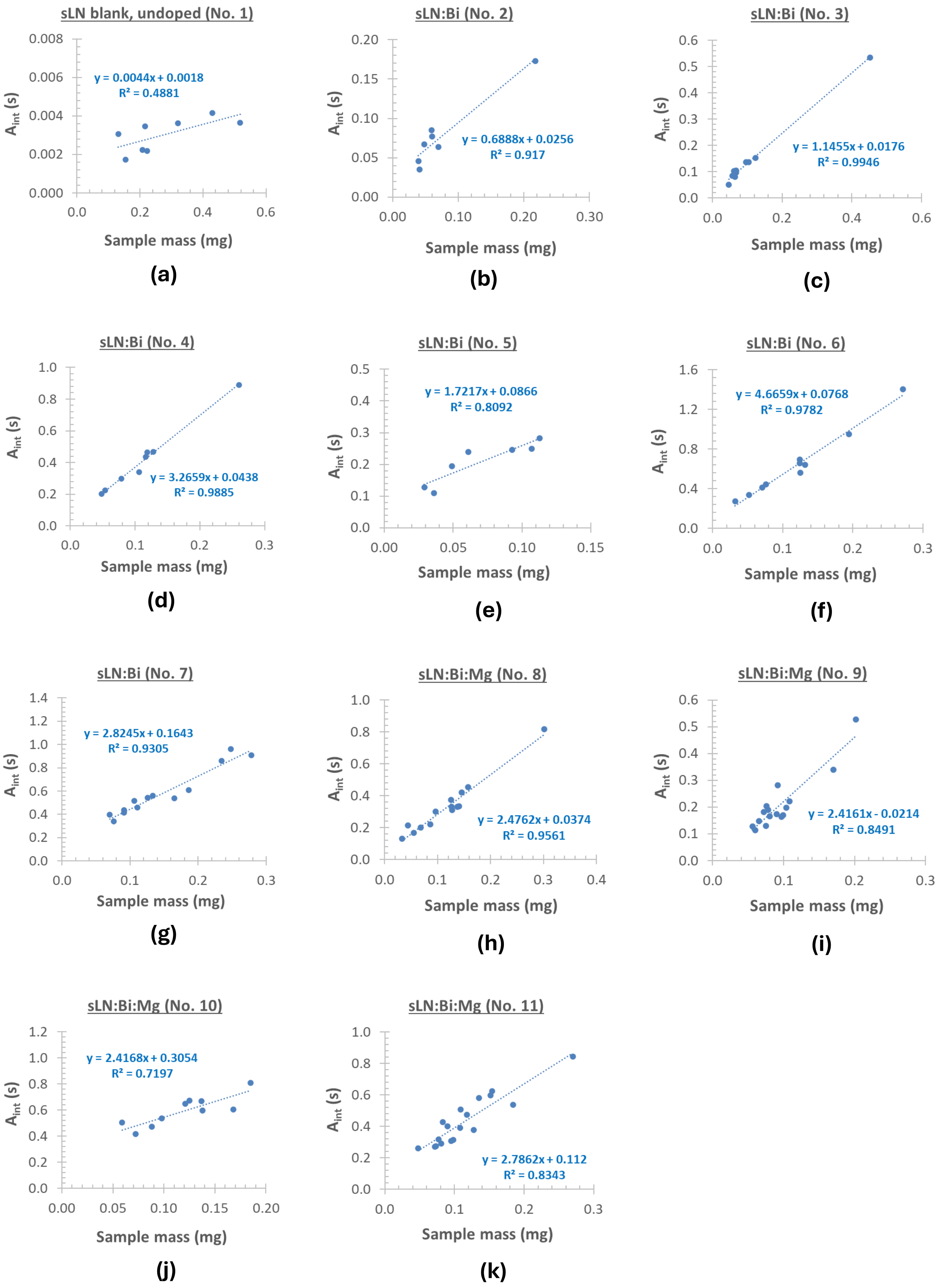

Supplement: Supplementary file 1 [file ao5c05486_si_001.pdf]
